# Ribavirin post-exposure prophylaxis for Andes virus exposure: a viewpoint

**DOI:** 10.1016/j.ebiom.2026.106386

**Published:** 2026-07-16

**Authors:** Roger J. Brüggemann, Albert Vollaard, Sacha de Stoppelaar, Johan G.C. van Hasselt, David M. Burger, Job J. Engel, Jeroen J.A. van Kampen, Matthew McCall, Loek Smits, Hetty Jolink, Leo G. Visser, Frank van de Veerdonk, Karin Ellen Veldkamp, Chantal P. Rovers, Rob J.W. Arts

**Affiliations:** a Department of Pharmacy, Pharmacology and Toxicology, and Radboudumc Community for Infectious Diseases, and Radboudumc Institute for Medical Innovation, Radboud University Medical Center, Nijmegen, the Netherlands; b Centre for Infectious Disease Control, National Institute for Public Health and the Environment (RIVM), Bilthoven, the Netherlands; c Department of Internal Medicine, Infectious Diseases Department, Amsterdam University Medical Center, Amsterdam, the Netherlands; d Leiden Academic Centre for Drug Research, Leiden University, Leiden, the Netherlands; e Department of Internal Medicine, and Radboud Institute of Medical Innovation (RIMI), Radboud University Medical Center, Nijmegen, the Netherlands; f Department of Viroscience, Erasmus MC, Rotterdam, the Netherlands; g Department of Medical Microbiology, Radboud University Medical Center, Nijmegen, the Netherlands; h Leiden University Center of Infectious Diseases, Subdepartment Infectious Diseases, Leiden University Medical Center, Leiden, the Netherlands; i Leiden University Center of Infectious Diseases, Subdepartment Medical Microbiology and Infection Control, Leiden University Medical Center, Leiden

**Keywords:** Hantavirus cardiopulmonary syndrome

## Abstract

Andes virus (ANDV) is unique among hantaviruses because person-to-person transmission is possible. This raises questions on the relevance of post-exposure prophylaxis (PEP), particularly following high-risk household, healthcare-associated, or laboratory exposures, considering its high case fatality rate. Ribavirin has demonstrated antiviral activity against hantaviruses *in vitro* and in animal models, although clinical evidence supporting its use for ANDV remains extremely limited. This viewpoint summarises the currently available evidence regarding ribavirin as PEP after potential ANDV exposure. We discuss the knowledge gaps that may limit the applicability of ribavirin PEP, to make informed decisions on the use of ribavirin in the setting of PEP. Potential dosing strategies are visualised by modelling and simulation, and potential dose and duration are discussed. Although the biological rationale for ribavirin PEP appears compelling, absence of controlled human studies and potential toxicity currently limit its role to highly selected exposure scenarios and use shortly after exposure.

## Introduction

Andes virus (ANDV) is one of the principal causes of hantavirus cardiopulmonary syndrome (HCPS) in South America, particularly in Chile and Argentina. It starts with a long incubation period, followed by a prodromal phase, finally resulting in HCPS, which is characterised by rapidly progressive capillary leak syndrome, pulmonary oedema, shock, and mortality rates frequently approaching 30–40%.^1^^,^^2^ Unlike most hantaviruses, ANDV has demonstrated person-to-person transmission, particularly among close household contacts and during exposure in the prodromal phase of infection.^3^, ^4^, ^5^ Household and healthcare-associated transmission in Argentina and Chile have been described.^3^^,^^4^ These observations support consideration of PEP after high-risk exposure. Despite this, the secondary attack rate after exposure appears to be relatively low overall. Most close contacts do not develop symptomatic infection even after prolonged or nosocomial exposure, with R_0_ of 2.12 (1.24–3.35).^5^ This complicates efforts to evaluate prophylactic efficacy. As a result, human studies to confirm either benefit or absence of benefit from PEP are difficult to conduct. The incubation period of ANDV infection is long, often ranging from two to four weeks, but up to six weeks has been described.^6^^,^^7^ This long preclinical phase theoretically creates a window of opportunity for antiviral intervention before the onset of general symptoms, and finally progression to HCPS. In addition, considering the low risk of infection, the antiviral used for PEP must have a high tolerability and safety, or else predominantly be used in well-defined high-risk categories.

Ribavirin, a guanosine analogue with broad-spectrum antiviral activity, has demonstrated activity against several RNA viruses, including arenaviruses and hantaviruses.^8^ So far, ribavirin has not shown therapeutic efficacy in the advanced state of HCPS and therefore its role in established HCPS remains at least controversial.^9^ Conversely, experimental animal models suggest that ribavirin may possess relevant antiviral activity during the asymptomatic and prodromal phase, before the onset of HCPS. Several other potential methods of PEP, such as neutralising monoclonal antibodies, are of potential interest but have not been developed yet. Only favipiravir has shown efficacy in hamster models, preventing disease when given within four days of experimental infection, and has been proposed for use as PEP.^10^ However, because no human dose recommendations have been established, and global access to favipiravir remains limited, the potential use of ribavirin as PEP warrants consideration.

Our viewpoint aims to synthesise the currently fragmented evidence on ribavirin as PEP for Andes virus, integrating *in vitro* findings, animal model data, and pharmacokinetic–pharmacodynamic considerations into a coherent framework.

By clarifying what is known, what remains uncertain, and which assumptions underlie proposed dosing strategies, the aim is to provide a transparent basis for evaluating ribavirin's potential role in high-risk exposure scenarios. These insights may support clinicians, public health physicians, and policymakers in making more informed, evidence-aligned decisions when confronted with possible ANDV transmission events.

## Search strategy and selection criteria

This viewpoint is informed by a non-systematic literature search on MEDLINE and PubMed using search terms “Hantavirus”, “Andes virus”, “ribavirin”, “Hantavirus cardiopulmonary syndrome” by an iterative approach, in which relevant publications were identified through reference lists, citation tracking, and related literature. All papers and abstracts or reports from meetings considered relevant to the scope of this viewpoint were reviewed and incorporated into the discussion. Only articles published in English and Spanish were included.

### *In vitro* evidence for ribavirin activity against ANDV

Experimental evaluation of ribavirin against ANDV has been performed using a Vero E6 cell culture system. Ribavirin inhibited ANDV replication *in vitro* in a concentration-dependent manner, reducing viral RNA and nucleoprotein synthesis.^11^^,^^12^ Inhibitory concentrations (IC50) ranged between 5 and 12.5 μg/mL (∼20–50 μmol/L), and 50% effective concentrations (EC50) 30 μg/mL, depending on the experimental conditions.^11^^,^^12^ Near-complete suppression of viral replication generally required higher concentrations, ranging from approximately 50–100 μg/mL.

### Animal studies supporting ribavirin PEP

The strongest evidence supporting ribavirin as PEP for ANDV derives from a Syrian hamster model, which remains the most widely accepted lethal animal model for HCPS.^13^ Safronetz et al. demonstrated that intraperitoneal administration of ribavirin (5, 25, 50, and 100 mg/kg/day for 10 days, 12 hamsters per dose group) administered 1 day after intraperitoneal inoculation of 100 × LD50 ANDV (154 focus forming units) provided substantial protection against lethal disease.^11^ All hamsters survived, whereas control animals (PBS alone) succumbed 8–10 days after infection, with only those in the 5 mg/kg/day group showing minor symptoms. Additional studies using 50 mg/kg/day showed that a 3-day regimen initiated on day 1 post-inoculation was fully protective, as was treatment starting on day 3. In contrast, initiating therapy on day 7—approximately one day before disease onset—resulted in nearly 100% mortality. Outcomes after oral treatment with both 5 and 50 mg/kg/day for 10 days starting one day post-inoculation were comparable to those with intravenous treatment, with only hamsters in the 5 mg/kg/day group showing minor symptoms.

Ogg et al. performed animal experiments in Syrian hamsters using lethal intranasal inoculation, which better resembles human infection through respiratory exposure, with a mean time to death of 14–26 days. Challenge with ANDV (4000 plaque forming units) while introducing ribavirin intraperitoneally at dosages ranging from 50 to 200 mg/kg/day from day 1–21 post inoculation was performed, to test for the capacity to prevent HCPS.^12^ While the highest dose of ribavirin (200 mg/kg) was toxic, both 100 and 50 mg/kg prevented HCPS in hamsters without serious adverse effects, whereas 7 out of 8 control-treated animals developed lethal HCPS. Treatment with 50 mg/kg/day ribavirin starting on days 6, 8, 10, or 12 post-infection also resulted in protection against HCPS in all groups. Administration of ribavirin at 14 days post-infection also still provided 75% protection against lethal HCPS.^12^

These hamster data provide a biological rationale for early ribavirin use as PEP in humans. Given that ANDV infection involves slow early viral replication and a long incubation period before clinical disease, a therapeutic window likely exists in the first days following high-risk exposure. As such, ribavirin PEP may prevent HCPS by suppressing viral replication before secondary immune-mediated endothelial injury develops,^14^ a pathogenic mechanism antiviral drugs cannot directly target.

The study by Ogg et al. (intranasal inoculation) indeed demonstrates a more prolonged effect of ribavirin as PEP compared with those by Safronetz et al. (intraperitoneal inoculation). This difference may be explained by the longer incubation period observed in the Ogg et al. model, which more closely resembles human infection and may allow more time for ribavirin to exert its prophylactic effect.

Compared with other viral haemorrhagic fevers, the available animal evidence supporting ribavirin PEP for ANDV is relatively strong. Nevertheless, important limitations remain, as animal models may not fully reproduce human pharmacokinetics, immune responses, or exposure dynamics (e.g. infectious dose), and the relationship between experimentally induced infection in animals and natural human exposure remains uncertain.

### Evidence for ribavirin post-exposure prophylaxis in humans

Human evidence supporting ribavirin as PEP after ANDV exposure is very limited. To date, no randomised controlled trials or prospective cohort studies have evaluated its efficacy in exposed individuals. Existing knowledge is therefore derived primarily from epidemiological investigations of person-to-person transmission and expert opinion (Supplementary Table S2). In a small study using a loading dose of 33 mg/kg, followed by 16 mg/kg, every 6 h for 15 doses, and concluding with 8 mg/kg every 8 h for 9 doses a positive effect on ANDV mortality was shown. Of seven patients treated with ribavirin during the prodromal phase, no fatalities were reported, compared to a historical case-fatality rate of 35%, and progression to the cardiopulmonary phase decreased from 84% to 57%.^15^

Evidence for therapeutic efficacy of intravenous ribavirin in new world hantavirus (Sin Nombre Virus) HCPS could not be established in a small RCT.^9^ The proportion of subjects who survived and who did not require ECMO was similar among the 10 ribavirin recipients and 13 placebo recipients (70% vs. 62%, respectively). Ribavirin recipients received treatment median 3.5 days (range 2–11) after onset of symptoms. Considering that ribavirin recipients progressed rapidly to either death or ECMO, median 4 h (range 3–15 h) after initiation of treatment, it was postulated that antiviral treatment was not effective after the onset of the cardiopulmonary phase. In this study the ribavirin dose was similar to that in an RCT which found ribavirin to be effective in the treatment of HFRS,^16^ which has a lower mortality than HCPS.

### Pharmacology of ribavirin

Ribavirin acts through several overlapping antiviral mechanisms, all occurring concurrently.^17^ Ribavirin is a nucleoside analogue which is intracellularly converted to mono-, di-, and triphosphate. Ribavirin-monophosphate is a competitive inhibitor of human inosine monophosphate dehydrogenase (IMPDH), disrupting *de novo* guanosine triphosphate (GTP) synthesis. Depletion of intracellular GTP pools starves the virus of nucleotides needed for replication. Ribavirin-triphosphate acts as a competitive inhibitor of RdRp. Incorporation of ribavirin-triphosphate stalls further viral RNA elongation and may cause chain termination. In addition, incorporation of ribavirin-triphosphate causes hypermutations in the subsequent rounds of viral replication and pushes the viral error rate beyond a survivable threshold, causing the viral population to collapse through accumulation of non-viable genomes.^18^, ^19^, ^20^ In parallel, ribavirin might exert immunomodulatory effects, by reducing ANDV-induced cytokine production.^21^

Ribavirin is rapidly and extensively absorbed after oral administration, with an average absolute bioavailability of ∼50–65% due to first-pass metabolism. Exposure increases linearly with dose for AUC across the 200–1200 mg range, while C_max_ shows non-linearity, tending to plateau above 400–600 mg, reflecting saturable processes in peak concentration but not overall exposure. Ribavirin relies heavily on saturable nucleoside transporters (ENT1/ENT2) in the gut for absorption. Fractionated dosing (e.g., 600 mg TID instead of 1200 mg BID) may improve absorption efficiency by avoiding transporter saturation. Bioavailability of a single oral dose of ribavirin was increased by co-administration with a high-fat meal.^22^, ^23^, ^24^, ^25^

Ribavirin is minimally protein-bound (<10%), meaning that nearly the entire circulating drug exists in the unbound, pharmacologically available form. In vitro studies indicate that ribavirin is not a substrate of CYP450 enzymes.^26^^,^^27^

Ribavirin pharmacokinetics are strongly affected by renal function because the drug is mainly eliminated by filtration and tubular excretion, while its intracellular metabolites have very long half-lives. Reduced kidney function decreases clearance, causing higher plasma and erythrocyte concentrations and increased trough levels, which are associated with toxicity, particularly haemolytic anaemia. Impaired renal clearance also delays steady state and prolongs elimination after treatment stops, which could even be beneficial during PEP. Clinically, patients with moderate to severe renal impairment have higher exposure at the same dose and often require dose reduction or avoidance.

### Toxicity and monitoring

Because of the severity of side effects anticipated, a balance must be struck between safety and invasive diagnostics in infected individuals in home quarantine and limited access to routine lab tests. Baseline evaluation before initiation of therapy generally includes complete blood count, renal function, liver enzymes, and pregnancy testing when relevant. During treatment, repeat haematologic, hepatic, and renal monitoring is typically performed approximately five to seven days after initiation, with additional (e.g. weekly) follow-up testing based on first results and clinical symptoms even after discontinuation of ribavirin, given its long half-life. Because of substantial teratogenic risk, ribavirin should be avoided during pregnancy.

Ribavirin-related anaemia is exposure-dependent. In chronic HCV, a quantitative relationship between red blood cell (RBC) RTP exposure, shortened RBC lifespan, and the time course and magnitude of haemoglobin decline has been demonstrated using population pharmacokinetic modelling. Simulations using this model indicate that reducing the standard 1000/1200 mg regimen to 600/800 mg can roughly halve the proportion of grade 2 anaemia, directly supporting a dose–toxicity relationship. Clinical risk factors that effectively increase exposure -impaired renal function, high dose per body weight, older age, and female sex-also increase anaemia risk, reinforcing the concentration-dependence of this toxicity.^22^^,^^24^ High upfront loading is expected to increase the likelihood and accelerate the onset of anaemia, even if the eventual nadir might be similar for a given total exposure.^27^^,^^28^

### Synthesis of findings to promote informed decision-making in the setting of PEP

No dosing regimen have been clinically evaluated for ribavirin PEP after ANDV exposure. Current regimens are extrapolated from other treatment protocols and expert-based protocols used for Lassa fever and Crimean-Congo haemorrhagic fever (Supplementary Tables S1 and S2), which both have shorter incubation times.^29^^,^^30^ A PEP strategy using augmented dosages should be carefully balanced against the high likelihood of toxicity and the low risk of human-to-human transmission.

The following aspects require careful consideration before clinical decisions can be made and will be discussed in the following paragraphs: 1. PK/PD considerations for ribavirin in relation to ANDV; 2. Dose of choice for PEP; and 3. Timing and duration of initiation of ribavirin therapy post contact.

### Ribavirin PKPD considerations for ANDV

Ribavirin does not have a single universally accepted PK/PD exposure target across viral pathogens, and for hantaviruses specifically, no definitive PK/PD exposure target has been established. Available studies focus on *in vitro* EC_50_/EC_90_ values and survival outcomes rather than formal PK/PD modelling. Ribavirin's mechanism— depletion of intracellular GTP pools, inhibition of RdRp, lethal mutagenesis and immunomodulation—makes its antiviral effect correlate poorly with extracellular plasma concentrations, complicating classical PK/PD index determination.

IC50/EC50 values are based on Vero E6 cell lines but may vary in other cell lines or different body tissues. Intracellular phosphorylation of ribavirin may be cell line dependent as has been observed for other nucleoside analogues.^31^ Thus, the intracellular concentration of ribavirin-triphosphate, and the degree of viral inhibition, may differ between cell types. This makes extrapolating the IC50/EC50 values found cell lines to the *in vivo* situation cumbersome.

If assumed that the Vero E6 cell line is best reflecting human conditions, the reported IC50 values for ANDV (5–12.5 μg/mL) can be reached by very aggressive dosing strategies with intravenous therapy. However, concentrations associated with near-complete suppression (50–100 μg/mL) of viral replication *in vitro* exceed concentrations that can be safely achieved in humans.^11^^,^^32^, ^33^, ^34^, ^35^ This raises uncertainty regarding the degree of antiviral suppression achievable *in vivo*. However, through extensive intracellular accumulation of phosphorylated ribavirin metabolites, intracellular concentrations substantially exceed plasma levels.^36^ Because hantavirus replication occurs intracellularly, intracellular pharmacodynamics, especially in macrophages and vascular endothelial cells which are primarily targeted by the virus, may be considerably more important than serum pharmacokinetics.^37^ This is further supported by the knowledge that IC50s of other viruses that are being treated with ribavirin have a comparable discrepancy between IC50 values and serum concentrations (Supplementary Table S3).

Directly translating intracellular accumulation from animal models to humans is not straightforward.^38^ Ribavirin enters intracellularly through nucleoside transporters and becomes trapped after phosphorylation to ribavirin-monophosphate, a metabolite that cannot exit the cell. This trapping mechanism exists across species, yet the extent and kinetics of accumulation differ markedly because cells vary in lifespan, transporter density, metabolic capacity, and intracellular kinase activity.^19^^,^^38^^,^^39^ These biological differences mean that while animal models can demonstrate the presence of intracellular trapping, they might not reliably predict the magnitude or time course of accumulation in humans. As a result, direct quantitative translation is not feasible.^29^^,^^30^

### Dose of choice for PEP

**Fig. 1:**
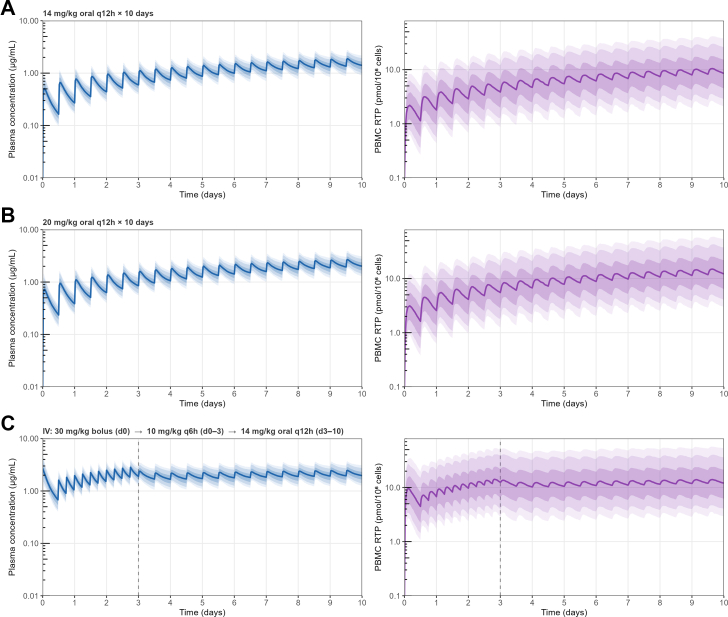
**Population pharmacokinetic simulation of ribavirin plasma concentrations and intracellular triphosphate accumulation in PBMCs under three dosing regimens.** Simulated ribavirin concentration–time profiles are shown for (A) 14 mg/kg oral, (B) 20 mg/kg oral, and (C) an IV-based loading regimen consisting of a 30 mg/kg IV bolus at day 0, followed by 10 mg/kg IV every 6 h from hour 12 through day 3, then switching to 14 mg/kg oral from day 3 to day 10. All regimens were simulated over a 10-day treatment course in a reference subject of 70 kg. Left panels show ribavirin plasma concentrations; right panels show intracellular ribavirin triphosphate (RTP) concentrations in peripheral blood mononuclear cells (PBMCs). The dashed vertical line in panel C indicates the transition from intravenous to oral administration. Simulations were performed using the population pharmacokinetic model reported by Wu et al. (2015),^43^ comprising a two-compartment plasma model with first-order oral absorption (assuming bioavailability F = 0.52 for oral dosing) which incorporated conversion of ribavirin to its phosphorylated forms in PBMCs. Lines represent the median and shaded regions the 10th–90th (dark) and 5th–95th (light) percentile intervals derived from N = 500 Monte Carlo simulated subjects.

When defining a dose for the use of PEP against ANDV upfront intravenous loading is warranted (Fig. 1) yet such an approach needs to be carefully balanced against the likelihood of treatment discontinuation due to severe adverse events.^8^^,^^29^^,^^40^, ^41^, ^42^

The most standardised dosing regimens for ribavirin currently used are those for hepatitis C and E, ranging from 10 to 15 mg/kg for several months to prevent chronic hepatitis (Supplementary Table S1). The difference with ANDV and other viral haemorrhagic fevers is that for hepatitis C/E no loading dose is required, as no instant effect is required, and stable blood concentrations can be achieved weeks later. In contrast, in ANDV and other viral haemorrhagic fevers, high upfront loading at dosages up to 35 mg/kg or 2.5 g/day have been advocated. Severe side effects are common at these dosages, although in the study of high dose intravenous ribavirin for 7 days in HFRS the drug-induced anaemia was rapidly reversible in all subjects.^16^

## Timing and duration of ribavirin PEP

Timing of initiation is critically important. It is unknown if partial suppression of early viral replication before onset of symptoms provides a clinically meaningful benefit, as high plasma viral loads before onset of symptoms have been reported.^7^ Partial suppression of early viral replication during the incubation phase could theoretically reduce viral dissemination, endothelial infection burden, and consequent cytokine production, contributing to an effect despite not attaining a specific IC50 target. Since severe HCPS most likely reflects immune-mediated injury, even modest attenuation of early viral amplification could beneficially affect the disease trajectory.^14^^,^^44^ This was demonstrated in the hamster model with the lowest ribavirin dose that modestly reduced viral titres but did result in reduction of mortality once HCPS had started.^11^ Because ribavirin is not driven by a single, direct, concentration-dependent mechanism, but instead emerges from several overlapping, time-dependent intracellular processes this may further support PEP. For many viruses, ribavirin's effect is mutagenic rather than inhibitory. This means that the antiviral effect depends on cumulative incorporation over time, not instantaneous concentration and that viral extinction occurs only after sufficient replication cycles have accumulated lethal mutations.

As brought forward earlier in this manuscript, experimental data strongly suggest that earlier treatment initiation is associated with greater efficacy.^11^^,^^12^ Ribavirin is therefore most likely to be beneficial when initiated within the first days after exposure, before viral dissemination and endothelial activation have occurred. Ogg et al. report that administration of ribavirin at 50 mg/kg/day starting on days 6, 8, 10, or 12 post-infection resulted in significant protection against HCPS in their hamster model with intranasal inoculation.^12^ No maximum interval for initiation of PEP can be deduced from animal studies: ribavirin administration up to 14 days after intranasal inoculation provided some clinical benefit in one study,^12^ but treatment after 7 days after intraperitoneal inoculation was not effective in another study.^11^

The optimal duration of prophylaxis remains unknown. Shorter courses may reduce cumulative toxicity and improve adherence, particularly if the primary therapeutic objective is attenuation of early viral replication. This is especially true, given ribavirin's long half-life of ∼150 h. Animal studies demonstrating protection administered ribavirin for 3–21 days of ribavirin during the incubation phase.^11^^,^^12^ Limited data have favoured ribavirin PEP treatment durations of approximately seven to ten days in high-risk Lassa fever virus exposures.^30^ This may allow for development of adaptive immunity, which was not aborted in hamsters treated with ribavirin.^44^ PEP for ANDV is expected to provide the greatest benefit when initiated very early after exposure, and this timing is likely more critical than the total duration of therapy.

## Proposal for ribavirin dosing schedule for ANDV PEP

Based on our assessment of current available preclinical and clinical evidence presented in this manuscript, we ideally propose a new dosing strategy, consisting of an intravenous loading dose of 30 mg/kg followed by 10 mg/kg IV bolus for 3 days, followed by 14 mg/kg oral up to day 10. This dosing schedule balances the need for high antiviral potency in the early phase with the risk of cumulative toxicity, with a finite 10-day course. An intensified early-phase strategy aims to achieve high systemic and intracellular ribavirin levels rapidly, thereby maximising antiviral pressure during the period when viral replication is still limited and before endothelial activation begins. In contrast, extending treatment over a longer period without an initial high-exposure phase would likely yield lower early drug levels, potentially reducing efficacy during the most biologically relevant window while increasing the risk of prolonged, dose-dependent adverse effects. To evaluate this dosing schedule from a PK/PD perspective, population PK modelling and simulation based on a previous published model in patients with HCV was performed.^43^ This model describes both the plasma PK of ribavirin as well as its intracellular phosphorylated forms in peripheral blood mononuclear cells (PBMCs), wherein the ribavirin triphosphate has been reported to be most strongly associated with antiviral activity. Importantly, ANDV does accumulate in PBMCs, given that buffy coats have a better performance for ANDV PCR detection than plasma.^45^ PK simulations were performed for both common oral dosing schedules of 14 and 20 mg/kg (Fig. 1A and B), as well as the proposed new dosing strategy (Fig. 1C). Our simulations illustrate how the proposed intensified intravenous treatment regimen can lead to a rapid achievement of intracellular RTP levels in comparison to a standard oral regimen.

## Practical considerations

Follow-up and management of side effects in individuals in quarantine can be challenging as it requires invasive diagnostic procedures on potentially infective samples. Sample management requires a laboratory that can deal with BSL3 pathogens. Routine follow-up of potentially exposed people includes at least weekly follow-up of PCR on whole blood as well as repeated serology testing.^46^ At these timepoints routine follow-up blood tests can also be performed to assess ribavirin-related toxicity, including complete blood count, hepatic, and renal monitoring. Based on the laboratory results over these 6 weeks it should be decided whether longer monitoring is required. Plasma concentrations of ribavirin will have decreased significantly at that point; yet intracellular metabolite concentrations have an even longer half-life, potentially causing anaemia after 6 weeks.

In theory ribavirin exposure, as represented at least by plasma levels, could be evaluated at these same time points as well. Whether plasma concentrations hold additional value can be debated, as there are no data on the correlation between plasma levels and intracellular concentrations of the active metabolites in the affected endothelial cells. Nor do plasma levels and IC50/EC50 values correlate well (Table 1 and Supplementary Table S3), which also holds true for other ribavirin treatment indications (Supplementary Tables S1 and S3). This suggests that plasma ribavirin levels are not a reliable correlate of treatment effect, so routine plasma concentration evaluations are not recommended. Although, to collect better data to answer these questions it should be considered to collect these data for research purposes. With this, emphasis of PK assessment should best focus on intracellular concentrations.

**Table 1:** Ribavirin plasma concentrations that can be achieved after several administration routes and doses.

Dose	Route	Plasma concentration	Ref
1000–1200 mg/day	Oral	2 μg/mL after one week, accumulating to 3.5 μg/mL after months	Jin R et al. *APSS J* 2012
1000–1200 mg/day	Oral	2.79 ± 0.80 μg/mL or 3.22 μg/mL ± 0.88 during steady state (after 9 weeks)	Wu LS et al. *Antimicrobial Agents and Chemotherapy* 2015
400 mg once	Oral I.v.	Cmax: 0.34 μg/mL 12.0 μg/mL	Gupta SK et al. Drug *Discoveries & Therapeutics* 2014
400 mg once	Oral	Cmax: 0.63 μg/mL	Gupta SK et al. *Drug Discoveries & Therapeutics* 2013
400–600 mg once	Oral	Cmax: 0.55 μg/mL	de Kanter CT et al. *Antivir Ther* 2015
1200 mg/day	Oral	After 12 weeks: Cmax: 2.7 ± 0.8 μg/mL Trough: 2.1 ± 0.8 μg/mL	FDA product label: https://www.accessdata.fda.gov/drugsatfda_docs/label/2005/021511s006lbl.pdf
100 mg/kg loading dose on the first day (up to a maximum of 7 g) in humans, followed by 25 mg/kg once daily from days 2–7 and 12.5 mg/kg once daily from days 8–10	Intravenous	95.1 ± 49.9 μg/mL, 32.4 ± 19.4 μg/mL, and 19.4 ± 10.9 μg/mL on sampling days 1, 4, and 10	Groger M et al. *Clin Infect Dis* 2023

### Putting knowledge into perspective to guide clinical decision making

The findings presented here highlight that ribavirin PEP for Andes virus exposure sits at the intersection of biological plausibility, encouraging preclinical data, and substantial clinical uncertainty. Arguments *in favour* of initiating PEP stem from the consistent protection observed in Syrian hamster models,^11^, ^12^, ^13^ the long incubation period of ANDV that creates a window of opportunity, and the finding in animal models that partial reduction of viral replication by low doses of ribavirin still resulted in clinical benefit. Furthermore, the high mortality of HCPS could outweigh potential adverse events. Conversely, arguments *against* routine PEP include the absence of human efficacy data, the inability to safely achieve fully suppressive concentrations observed *in vitro*,^11^ and the well-recognised toxicity of ribavirin—particularly haemolytic anaemia—which becomes harder to justify in low-probability exposure scenarios. Taken together, these considerations suggest that PEP should be reserved for clearly defined high-risk contacts, such as needle stick accidents, unprotected mucosal exposure, prolonged exposure as in household contacts, or documented breaches in laboratory or healthcare procedures. A case-by-case consideration should be made based on type of exposure, duration, and viral loads in the specific specimen. If PEP is to be initiated, this should happen as soon as possible after exposure, ideally within the first days when viral amplification is still limited, though we acknowledge that the precise outer boundary of effectiveness in humans remains unknown.

An important conceptual issue is that complete viral suppression may not be necessary for clinical benefit. Firstly, reduced and delayed viral replication allows for more time to mount an effective immune response to neutralise the virus. Secondly, since HCPS likely reflects secondary immune-mediated endothelial injury and partial attenuation of early viral amplification could theoretically reduce downstream inflammatory injury even in the absence of sterilising antiviral activity, resulting in milder disease.

Taking all presented considerations into account and combining these with our simulation data we propose a PEP scheme of 10 days, which should be considered as an accelerated loading phase. Ideally, this would be started with an intravenous loading phase before continuing oral loading of 14 mg/kg divided over 2 doses and taken with food. However, given the issues of intravenous loading, an all-oral loading phase with 20 mg/kg divided over 2 doses could also be considered (Fig. 1). In this way, plasma levels of >1 μg/mL could be achieved directly, or within a few days, by using a dose which is 1.5–2 times higher than those used in hepatitis C/E treatment, hence optimising the prophylactic effect and potential adverse drug effects.

This framework could enable clinicians to make informed, transparent decisions that weigh biological rationale, exposure risk, and patient safety in the absence of definitive human data. It is therefore essential that when PEP is started, follow-up data should be carefully monitored and shared with the scientific community to further substantiate future decisions.

## Outstanding questions

Future research should prioritise translational studies that bridge the gap between promising animal data and clinical application. Given the rarity of person-to-person transmission, prospective international registries of high-risk exposures may provide the most feasible approach to evaluating the effectiveness and safety of ribavirin PEP. Standardised collection of exposure characteristics, timing of treatment initiation, dosing regimens, pharmacokinetic data, adverse events, and clinical outcomes would substantially strengthen the current evidence base and is most likely the highest evidence that can be collected.

Further refinement of animal models is also warranted. Existing Syrian hamster studies have demonstrated that ribavirin retains efficacy when initiated several days after infection, but systematic evaluation of delayed treatment, different dosing regimens, oral versus intravenous administration, and especially combination treatment with other antiviral drugs, or assessment of other potentially more potent antiviral drugs is warranted. Importantly, more clinically relevant models, such as a human lung xenograft murine model^47^ are now available to assess these questions.

Equally important is the need for improved pharmacokinetic and pharmacodynamic (PK/PD) characterisation of ribavirin in the setting of hantavirus infection. Future studies should define the relationship between plasma concentrations, intracellular phosphorylated ribavirin metabolites, and antiviral activity against ANDV. Finally, mathematical modelling integrating viral kinetics, intracellular drug disposition, and experimentally derived inhibitory concentrations may allow simulation of human dosing strategies capable of achieving antiviral exposures comparable to those associated with protection in animal models. Such modelling could inform evidence-based selection of oral or intravenous dosing regimens before clinical implementation. Collectively, these studies would provide the mechanistic and translational evidence required to determine whether ribavirin PEP can become a rational and effective intervention for high-risk exposures to ANDV.

## Contributors

RJB and RJWA draughted the original manuscript, JGCvH performed the modelling, all others reviewed and improved the manuscript. All authors read and approved the final version of the manuscript.

## Declaration of interests

None for this manuscript.
