# Supplementary material for: Ribavirin post-exposure prophylaxis for Andes virus exposure: a viewpoint
Source: eBioMedicine. 2026 Jul 16;130:106386. doi: 10.1016/j.ebiom.2026.106386 (PMC13400777; doi:10.1016/j.ebiom.2026.106386)
Supplement: Supplementary Tables S1–S3 [file mmc1.docx]

**Supplementary Table 1 – Routine ribavirin dosing schemes**

| **Viral disease** | **Route** | **Typical adult dosing regimen** | **Usual duration** | **Key notes** |
| --- | --- | --- | --- | --- |
| Chronic hepatitis C (historical, combo) | Oral | 800–1,200 mg/day in divided doses (weight‑based: ~1,000 mg \<75 kg; 1,200 mg ≥75 kg) | 24–48 weeks | Given with peg‑IFN; anemia‑limited; now largely replaced by DAAs. |
| RSV (severe, mainly infants/ICU) | Inhaled | 20 mg/mL solution via small‑particle aerosol, 12–18 hours/day (continuous or intermittent) | 3–7 days | Used in high‑risk severe RSV; specialized administration. |
| Lassa fever | IV | 30 mg/kg loading, then 16 mg/kg/day (often divided BID) | 10 days (common regimen) | Early initiation critical; dosing varies by protocol. |
| Crimean–Congo hemorrhagic fever (CCHF) | Oral/IV | Oral: 2 g loading, then 1 g Q6H day 1, then 500 mg Q6H days 2–5 | 5–10 days | Regimens differ; evidence mixed but used in endemic settings. |
| Hantavirus (HFRS/H**C**PS) | Oral/IV | Similar to CCHF: high loading (e.g., 2 g), then 1 g Q6H day 1, then 500 mg Q6H days 2–5 | 5–10 days | Benefit clearer in HFRS if given early; limited data in HCPS. |
| Chronic hepatitis E (off‑label) | Oral | 600–1,000 mg/day in divided doses (often ~600–800 mg/day) | 3 months (often extended if needed) | Used mainly in immunocompromised; dose adjusted for renal function and anemia. |
| SARS‑CoV‑1 / MERS / early COVID‑19 (past) | Oral/IV | Highly variable; oral often 1,200–2,400 mg/day in divided doses; IV high‑dose in some trials | 7–14 days (trial‑dependent) | Now largely abandoned; toxicity and lack of clear benefit at high doses. |

**Supplementary Table 2 – Ribavirin used as PEP**

| **Virus** | **Model** | **Ribavirin Route** | **Representative Dosing Regimen** | **Latest Effective Start After Infection** | **Main Findings** | **Reference** |
| --- | --- | --- | --- | --- | --- | --- |
| Lassa virus | Nonhuman primates | Intravenous | 50 mg/kg loading dose followed by 10 mg/kg every 8 h for 18 days | Up to 4 days | Protected against fatal infection; efficacy improved with early treatment | Jahrling PB et al. *J Infect Dis* 1980^1^ |
| Lassa virus | 25 humans with high exposure | Oral | 400mg twice daily for 10 days | <2 days | No infections | Hadi CM et al. *Emerg Infect Dis* 2010^2^ |
| Lassa virus | 16 humans with high exposure | Oral | 10 mg/kg 4 times daily for 5-8 days | ? | No infections | Haas WH et al. *Clin Infect Dis* 2003 ^3^ |
| Lassa | 7 humans with high exposure | oral | 600mg 4 times daily for 10 days |  | No infections | Holmes GP et al. *NEJM* 1990^4^ |
| CCHF | 28 humans with high exposure | Oral | 1600mg loading dose, then 500mg three times daily for 5 days |  | No infections | Saleem J et al. *Int J Infect Dis* 2009^5^ |
| Junin virus | Rhesus macaques | Intravenous | 60 mg/kg for 4 days, then 30 mg/kg for 3.5 days, then 15 rng/kg for 11 days | Up to 6 days | Protection against mortality | Weissenbacher et al. *J Med Virol* 1986^6^ |
| Andes virus | Syrian hamsters | Intraperitoneal | 5–100 mg/kg/day for 10 days | Up to 3 days | Significant survival benefit and reduced viral replication | Safronetz D et al. *PLoS One* 2011^7^ |
| Andes virus | Syrian hamsters (intranasal challenge) | Intraperitoneal | 50-100mg/kg/day for 21 days | Up to 14 days | Significant survival benefit and reduced viral replication | Ogg M et al. *Viruses* 2013^8^ |
| **Reviews** | | | | | | |
| Lassa virus | Review | oral | a 35-mg/kg loading dose (maximum dose, 2.5 g) followed by 15 mg/kg (maximum dose, 1 g) 3 times a day for 10 days |  |  | Bausch DG et al. *Clin Infect Dis* 2010^9^ |
| Crimean- Congo hemorrhagic fever | Systematic review meta analysis | Oral | Different regimens, most 2-2.4g/day with reduction to 1.2g/day after 3-4 days |  | PEP reduced the odds of infection (OR 0.01, 95% CI 0–0.03), and ribavirin use <48 hours after symptom onset reduced the odds of death (OR 0.03, 95% CI 0–0.58). | Ergönül O et al. *Emerg Infect Dis* 2018^10^ |

**Supplementary Table 3 - Reported IC50/EC50 values**

| **Virus** | **Experimental System** | **IC50** | **EC50** | **Units** | **Key Reference** |
| --- | --- | --- | --- | --- | --- |
| Andes hantavirus | Vero E6 cells | 5–12.5 | — | μg/mL | Safronetz D et al. *PLoS One.*2011^7^ |
| Andes hantavirus | Vero E6 cells | — | 30 | μg/mL | Ogg M et al. *Viruses* 2013^8^ |
| Hantaan virus | Vero E6 cells | — | 14.8 | μg/mL | Kirsi JJ et al. *Antimicrob Agents Chemother* 1983^11^ |
| Lassa fever | Cell culture systems | 9-20 | 9-20 | μg/mL | Summarized by Bausch DG et al. *Clin Infect Dis* 2010^9^ |
| CCHF | Huh7 cell line |  | 3 | μg/mL | Welch SR et al. *Antiviral Res* 2017^12^ |
| Hepatitis E | Hepatoma cell lines | — | 0.7 | μg/mL | Debing Y et al. *Antimicrob Agents Chemother* 2014 *^13^* |
| Hepatitis C | HCV replicon model | 2.9–36.5 | — | μg/mL | Lau JYN et al. *Hepatology* 2002^14^ |

**References**

1. Jahrling PB, Hesse RA, Eddy GA, Johnson KM, Callis RT, Stephen EL. Lassa virus infection of rhesus monkeys: pathogenesis and treatment with ribavirin. *J Infect Dis* 1980; **141**(5): 580–9.

2. Hadi CM, Goba A, Khan SH, et al. Ribavirin for Lassa fever postexposure prophylaxis. *Emerg Infect Dis* 2010; **16**(12): 2009–11.

3. Haas WH, Breuer T, Pfaff G, et al. Imported Lassa fever in Germany: surveillance and management of contact persons. *Clin Infect Dis* 2003; **36**(10): 1254–8.

4. Holmes GP, McCormick JB, Trock SC, et al. Lassa fever in the United States. Investigation of a case and new guidelines for management. *N Engl J Med* 1990; **323**(16): 1120–3.

5. Saleem J, Usman M, Nadeem A, Sethi SA, Salman M. Crimean-Congo hemorrhagic fever: a first case from Abbottabad, Pakistan. *Int J Infect Dis* 2009; **13**(3): e121–3.

6. Weissenbacher MC, Calello MA, Merani MS, McCormick JB, Rodriguez M. Therapeutic effect of the antiviral agent ribavirin in Junin virus infection of primates. *J Med Virol* 1986; **20**(3): 261–7.

7. Safronetz D, Haddock E, Feldmann F, Ebihara H, Feldmann H. In vitro and in vivo activity of ribavirin against Andes virus infection. *PLoS One* 2011; **6**(8): e23560.

8. Ogg M, Jonsson CB, Camp JV, Hooper JW. Ribavirin protects Syrian hamsters against lethal hantavirus pulmonary syndrome--after intranasal exposure to Andes virus. *Viruses* 2013; **5**(11): 2704–20.

9. Bausch DG, Hadi CM, Khan SH, Lertora JJ. Review of the literature and proposed guidelines for the use of oral ribavirin as postexposure prophylaxis for Lassa fever. *Clin Infect Dis* 2010; **51**(12): 1435–41.

10. Ergonul O, Keske S, Celdir MG, et al. Systematic Review and Meta-analysis of Postexposure Prophylaxis for Crimean-Congo Hemorrhagic Fever Virus among Healthcare Workers. *Emerg Infect Dis* 2018; **24**(9): 1642–8.

11. Kirsi JJ, North JA, McKernan PA, et al. Broad-spectrum antiviral activity of 2-beta-D-ribofuranosylselenazole-4-carboxamide, a new antiviral agent. *Antimicrob Agents Chemother* 1983; **24**(3): 353–61.

12. Welch SR, Scholte FEM, Flint M, et al. Identification of 2'-deoxy-2'-fluorocytidine as a potent inhibitor of Crimean-Congo hemorrhagic fever virus replication using a recombinant fluorescent reporter virus. *Antiviral Res* 2017; **147**: 91–9.

13. Debing Y, Emerson SU, Wang Y, et al. Ribavirin inhibits in vitro hepatitis E virus replication through depletion of cellular GTP pools and is moderately synergistic with alpha interferon. *Antimicrob Agents Chemother* 2014; **58**(1): 267–73.

14. Lau JY, Tam RC, Liang TJ, Hong Z. Mechanism of action of ribavirin in the combination treatment of chronic HCV infection. *Hepatology* 2002; **35**(5): 1002–9.
